# Supplementary material for: Vitamin C Maintenance against Cell Growth Arrest and Reactive Oxygen Species Accumulation in the Presence of Redox Molecular Chaperone hslO Gene
Source: Int J Mol Sci. 2022 Oct 24;23(21):12786. doi: 10.3390/ijms232112786 (PMC9659236; doi:10.3390/ijms232112786)
Supplement: Supplementary file 1 [file ijms-23-12786-s001.zip › ijms-1938551-supplementary.pdf]

International Journal of *Molecular Sciences*

Supplementary Materials

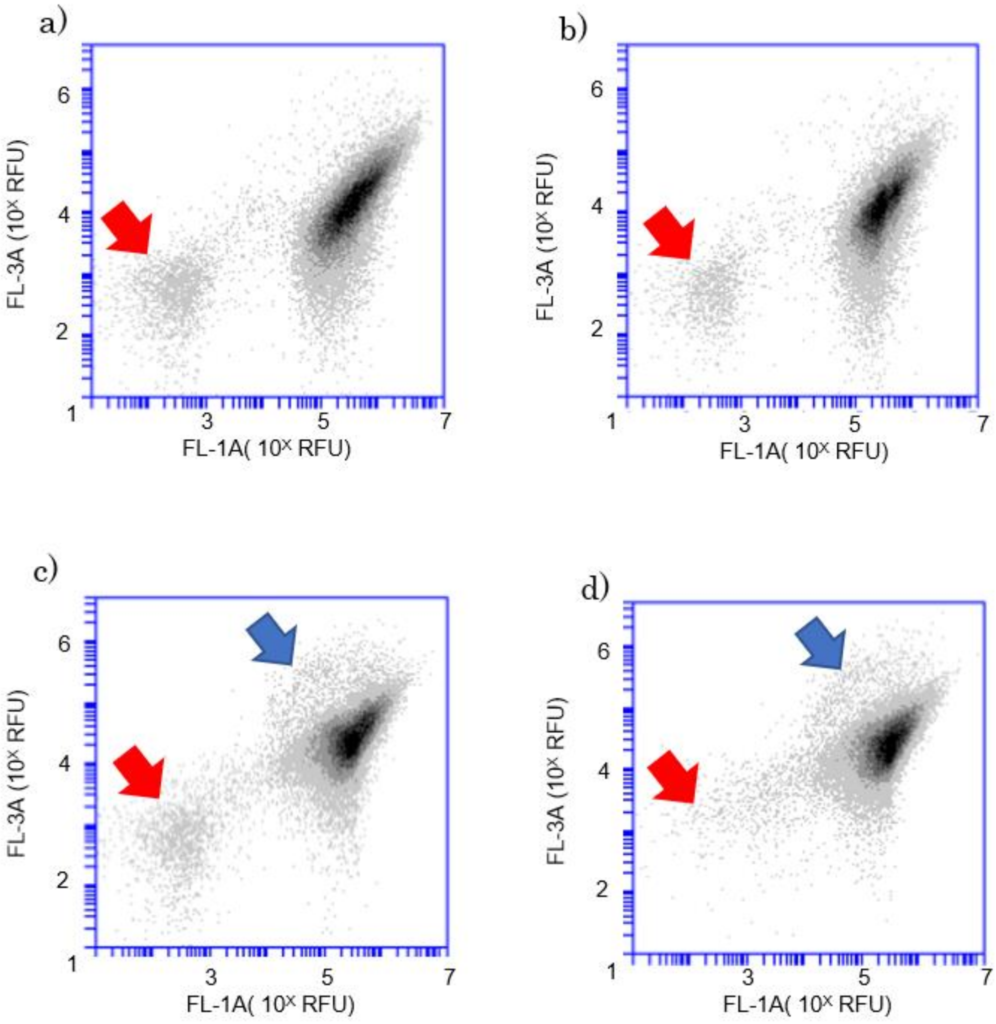

Figure S1. *recAts polA and recAts polA ΔhslO* cells analyzed with live-dead staining at the restricted and permissive temperatures. (**a**) *recAts polA* cells analyzed with live-dead staining at the permissive temperature. TK3077 (*recAts polA*) cells cultivated at 30 °C, stained with live-dead staining. The x-axis represents the FL-1-A: SYTO9-stained DNA contents of the cell, and the y-axis represents the FL-4A: PI-stained DNA contents of the cell. The red arrows indicate the position of cell populations which possessed very low DNA contents. The results of 10,000 particles are shown. (**b**) *recAts polA ΔhslO* cells analyzed with live-dead staining at the permissive temperature. TK3276 (*recAts polA ΔhslO*) cells cultivated at 30 °C and stained as indicated in (a). (**c**) *recAts polA* cells analyzed with live-dead staining at restricted temperature. TK3077(*recAts polA*) cells cultivated at 42°C and stained as indicated in (**a**). The blue arrows indicate the positions of membrane-damage dead-cell populations. (**d**) *recAts polA ΔhslO* cells analyzed with live-dead staining at the restricted temperature. The TK3276 (*recAts polA ΔhslO*) cells were cultivated at 42 °C and were stained as indicated in (c).

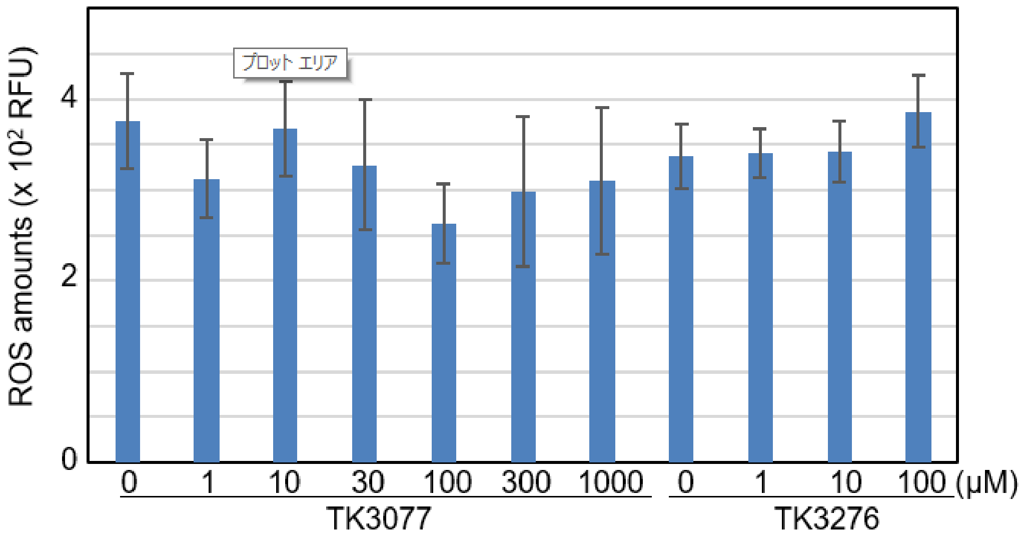

Figure S2. Effect of vitamin C on mean reactive oxygen species (ROS) levels of *recAts polA* cells and *recAts polA ΔhslO* at restricted conditions. In the early logarithmic growth phase (O.D.$_{600}$ = 0.1), the TK3077 (*recAts polA*) and TK3276 (*recAts polA ΔhslO*) cultures were divided into four portions, and each was incubated at 42 °C either with or without the indicated vitamin C concentration on the horizontal axis. Each bar represents the average and standard error of mean: SEM (n ≥ 3).

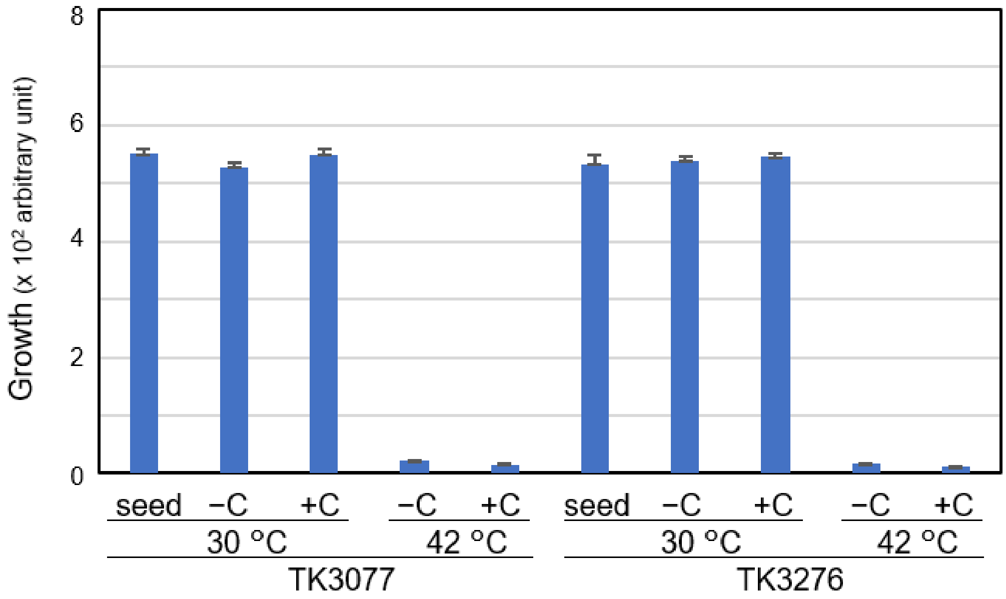

Figure S3. Effect of vitamin C on the growth of *recAts polA* cells and *recAts polA ΔhslO* at permissive and restricted conditions with the inoculation experiments. TK3077 (*recAts polA*) and TK3276 (*recAts polA ΔhslO*) cells were inoculated with 1/100 of the overnight seed culture and incubated either with or without 100 μM vitamin C at 30 °C or 42 °C for 16 h. Growth of cells determined as measures in turbidity (O.D.$_{600}$ arbitrary unit) after the culture medium was divided. The growth of the seed and cultured cells at 30 °C or 42 °C for 16 h were compared. In the figure, a combination of the bars is represented from left to right: seed culture, cell culture without viatmin C (−C), or with 100 μM vitamin C (+C) at both 30 °C and 42 °C for TK3077 and TK3276 cells. Each bar represents the average and standard error of mean: SEM (n ≥ 7).

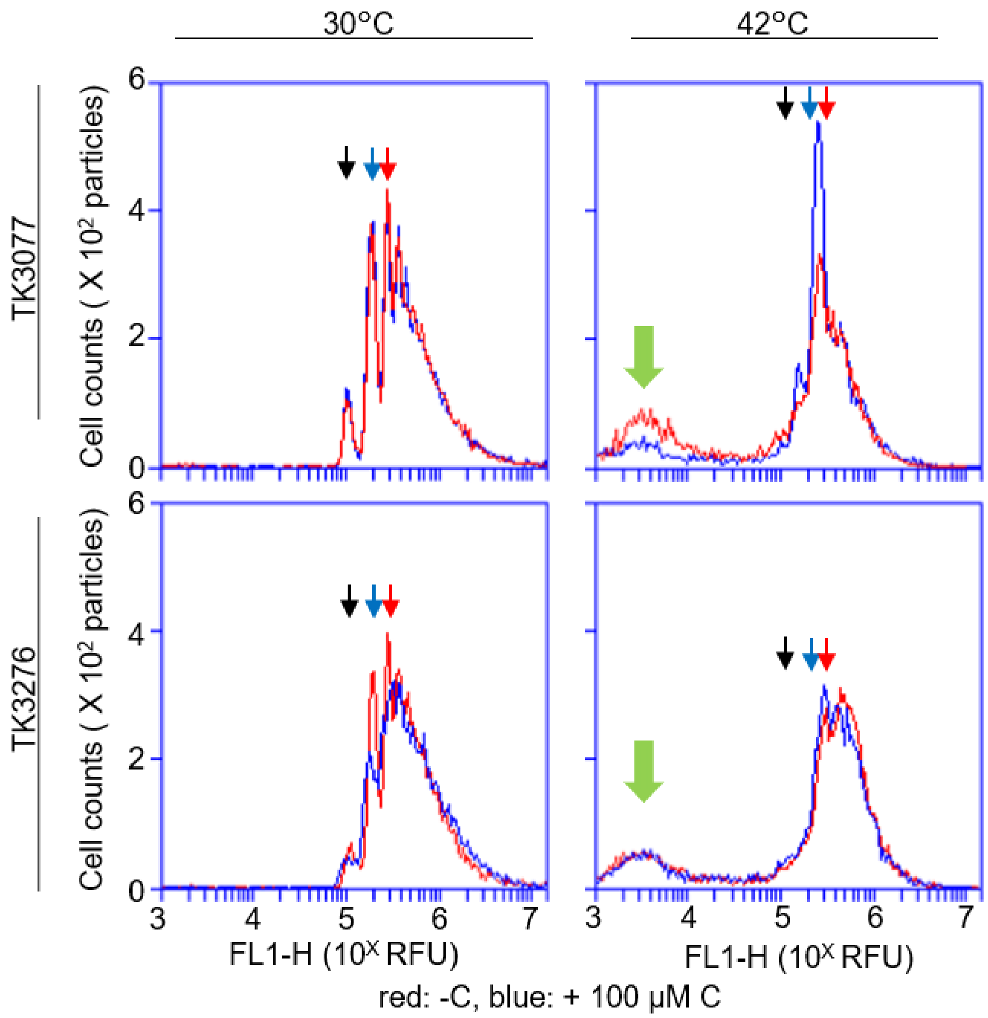

Figure S4. Chromosome content comparison in restrictive and permissive conditions for the *recAts polA* and *recAts polA ΔhslO* cells. The DNA of TK3077 (*recAts polA*) and TK3276 (*recAts polA ΔhslO*) cells was quantitatively compared between permissive and restrictive temperatures with or without 100 µM vitamin C. Either TK3077 (upper) or TK3276 (lower), either 30 °C (left) or 42 °C (right), and either without vitamin C (red) or with 100 µM vitamin C (blue) are shown. A total of 20,000 particles are shown. Arrowheads indicate ploidies as 1 (black), 2 (red), and 3 (blue). Thick green arrowheads indicate a position of anucleate cells.

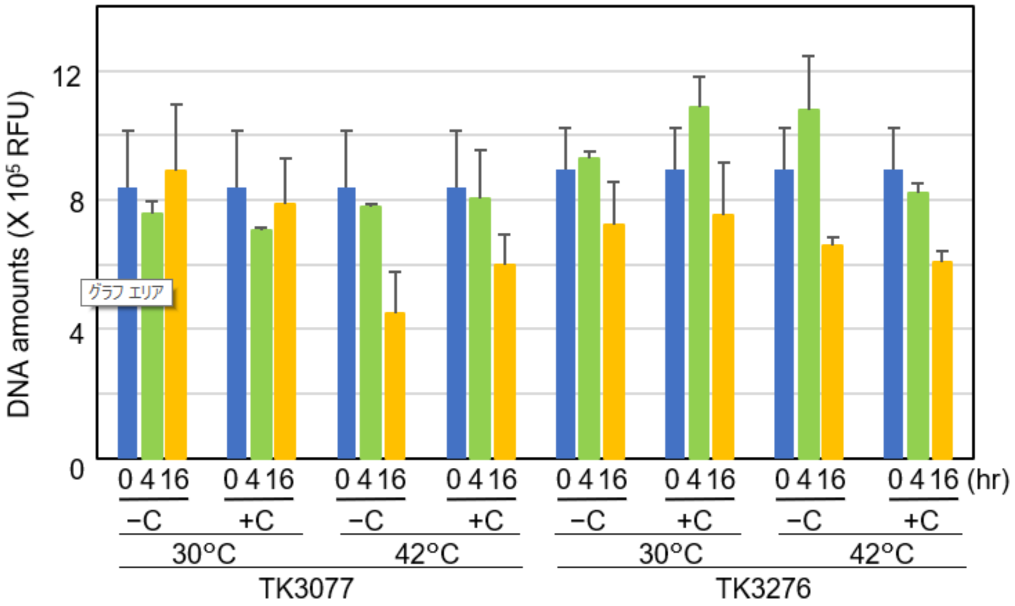

Figure S5. Effects of vitamin C on the DNA quantity in *recAts polA* cells and *recAts polA ΔhslO* cells. In the early logarithmic growth phase (O.D.$_{600}$ = 0.1), the TK3077 (*recAts polA*) and TK3276 (*recAts polA ΔhslO*) cultures were divided into four portions, and each was incubated either with or without vitamin C at 30 °C and 42 °C, at start time 0. The DNA quantities in both TK3077 and TK3276 cells were quantitatively measured using PicoGreen. The DNA quantity was determined using the mean FL-1A with the P3 gate. The chronologically arranged samples are shown as time 0 (blue), 4 h (green), and 16 h (yellow). Strains, temperature, and vitamin C are demonstrated below the graph. Each determination is presented as the average and standard error of the mean (n ≥ 3, SEM).

Table S1. ***Escherichia coli*** **strains used in this study**

**Strain**	**Relevant Genotype**	**Source, Reference, or Construct**
AQ634		This laboratory
AQ1025	*lexA51 malB*::Tn*9*	This laboratory
AQ6730	KL268 *recA200*	This laboratory
AQ8534	*polA25*::*spc zih-35*::Tn*10*	This laboratory [37]
AQ10459	As AQ10458	duplicated stock for AQ10458 [3]
AQ10546	AQ10458 *recA200*	This laboratory [2]
AQ10549	AQ10546 *polA25 malF*::Tn*10*	This laboratory [2]
AQ10865	*lexA51 malB*::Tn*9*	This laboratory
AQ11369	*ΔhslO*::*Km*	This laboratory [2]
TK3019	AQ634 *malB*::Tn*9 lexA*$^{+}$	AQ634 x P1.AQ1025 Cm^{r}, Tr
TK3077	As AQ10549	AQ10549 regrow
TK3276	AQ10549 *ΔhslO*::*Km*	AQ10549 x P1.AQ11369 Km^{r}, PCR
TK3473	TK3276 *p*EXvec	TK1219 × *p*EXvec → Ap^{r}
TK3474	TK3276 *p*EX*hslO*	TK1217 ×*p*EX*hslO* → Ap^{r}

Tr, temperature-resistant growth at 42 °C; Ts, temperature-sensitive growth at 42 °C; Tc^{r}, Km^{r}, Cm^{r}, and Ap^{r} denote resistance to tetracycline, kanamycin, chloramphenicol, and ampicillin, respectively; Tc^{s}, Km^{s}, and Cm^{s} denote susceptibility to tetracycline, kanamycin, and chloramphenicol, respectively; UV^{s}, sensitive to UV; UV^{r}, resistant to UV.

AQ634: genotype: *trpA9605 his-29 proB*(*or proC*) *ilv metB1 thyA deoB*(*or deoC*)

AQ10458 genotype: *F- argE3 leuB-6 proA2 thr-1 thi-1 rpsL31 galK2 lacY1 mtl-1 supE44 sfiA11*
